# Construction of diagnostic prediction model for Wilson's disease

**DOI:** 10.3389/fsurg.2022.1065053

**Published:** 2023-01-05

**Authors:** Yao Wang, Yulian Li, Linting Xun, Zhengji Song

**Affiliations:** ^1^ Medical Faculty of Kunming University of Science and Technology, Affiliated with the First People's Hospital of Yunnan Province, Kunming, China; ^2^ Department of Gastroenterology, The First People's Hospital of Yunnan Province, Kunming, China

**Keywords:** Wilson’s disease, diagnostic prediction model, serological indicators, Chinese patients, nomogram prediction model

## Abstract

**Background:**

Wilson's disease, also known as hepatolenticular degeneration, is a rare human autosomal recessive inherited disorder of copper metabolism. The clinical manifestations are diverse, and the diagnosis and treatment are often delayed. The purpose of this study is to establish a new predictive diagnostic model of Wilson's disease and evaluate its predictive efficacy by multivariate regression analysis of small trauma, good accuracy, low cost, and quantifiable serological indicators, in order to identify Wilson's disease early, improve the diagnosis rate, and clarify the treatment plan.

**Methods:**

A retrospective analysis was performed on 127 patients with Wilson's disease admitted to the First People's Hospital of Yunnan Province from January 2003 to May 2022 as the experimental group and 73 patients with normal serological indicators who were not diagnosed with Wilson's disease. SPSS version 26.0 software was used for single factor screening and a multivariate binary logistic regression analysis to screen out independent factors. R version 4.1.0 software was used to establish an intuitive nomogram prediction model for the independent influencing factors included. The accuracy of the nomogram prediction model was evaluated and quantified by calculating the concordance index (C-index) and drawing the calibration curve. At the same time, the area under the curve (AUC) of the nomogram prediction model and the receiver operating characteristic (ROC) curve of the Leipzig score was calculated to compare the predictive ability of the nomogram model and the current Leipzig score for Wilson's disease.

**Results:**

Alanine aminotransferase (ALT), aspartate aminotransferase (AST), alkaline phosphatase (AKP), albumin (ALB), uric acid (UA), serum calcium (Ca), serum phosphorus (P), and hemoglobin (HGB) are closely related to the occurrence of Wilson's disease (*p *< 0.1). The prediction model of Wilson's disease contains seven independent predictors: ALT, AST, AKP, ALB, UA, Ca, and P. The AUC value of the prediction model was 0.971, and the C-index value was 0.972. The calibration curve was well fitted with the ideal curve. The nomogram prediction model had a good predictive effect on the occurrence of Wilson's disease; the ROC curve of Leipzig score was drawn, and the AUC value was calculated. The AUC of the Leipzig score was 0.969, indicating that the prediction model and the scoring system had predictive value, and the nomogram prediction model had a better predictive effect on the research objects of the center.

**Conclusion:**

ALT, AST, AKP, ALB, UA, Ca, and P are independent predictors of Wilson's disease, and can be used as early predictors. Based on the nomogram prediction model, the optimal threshold was determined to be 0.698, which was an important reference index for judging Wilson's disease. Compared with the Leipzig score, the nomogram prediction model has a relatively high sensitivity and specificity and has a good clinical application value.

## Introduction

Wilson's disease (WD) was first described in 1912 by Dr Samuel Alexander Kinnier Wilson, which is a rare human autosomal recessive inherited disorder of copper metabolism. The prevalence is approximately 3 in 100,000, and the carrying rate of pathogenic genes is about 1 in 90 (1). WD is caused by a mutation in the *ATP7B* gene, which is located on 13q14.3. *P*-type copper-transporting ATPase is the product of this gene, which consists of six metal-binding domains and eight transmembrane domains, making copper dependent on ATP transport on the membrane (2). This protein has two specific functions in liver cells. First, it provides copper to the trans-Golgi network (TGN) to bind to major copper transporters secreted into the blood. Second, it promotes the excretion of excess copper by transferring copper to a late lysosomal compartment and isolating it into vesicles for export through the apical tubule membrane (3). *ATP7B* gene mutation weakens or loses the function of ATPase, resulting in the decreased synthesis of ceruloplasmin in serum and copper excretion disorder, which makes copper ions accumulate in the liver, brain, kidney, and cornea, and lacks specificity.

WD can occur at any age, and liver damage occurs in children aged 10-13 years (4). It can manifest as asymptomatic hepatomegaly, a continuous increase of liver enzymes, acute or chronic hepatitis, compensatory or decompensated cirrhosis, and even fulminant liver failure. Neuropsychiatric symptoms are more common in patients aged 10–30 years (5, 6), manifesting as involuntary shaking of the limbs, dystonia, limb stiffness and bradykinesia, and mental and behavioral abnormalities. Although the disease is rare, it is called ‘not rare rare disease’. Higher incidence in China, more common in adolescents, more men than women.

At present, the early clinical manifestations of WD are not typical, and the related examinations and genetic tests are more complicated. Therefore, it is necessary to explore the combined detection of multiple serological indicators as a new idea. The nomogram prediction model is a visual, intuitive, and convenient statistical model, which provides predictive information for clinicians as a clinical tool. Therefore, constructing a nomogram prediction model can help to diagnose early WD. The purpose of this study is to explore the risk factors of WD in a Chinese population and to explore possible predictive models.

## Participants and methods

### Participants

The clinical data of 127 patients with Wilson disease (experimental group ) and 73 subjects with normal serological indicators admitted to the First People's Hospital of Yunnan Province from January 2003 to May 2022 were retrospectively analyzed. After determining the inclusion and exclusion criteria, the clinical data of 200 patients were included.

### Inclusion and exclusion

#### Inclusion criteria

The inclusion criteria were determined according to the Leipzig scoring criteria issued by the European Liver Association in 2012 (patients with WD).

##### non-WD patients included in the normal serological subjects

The normal serological indicators of undiagnosed WD may be associated with unexplained neuropsychiatric symptoms or decreased ceruloplasmin, and so on (non-WD patients).

#### Exclusion criteria

The exclusion criteria were as follows: (1) patients with uneven clinical data; (2) patients with liver damage (viral liver disease, alcoholic hepatitis, autoimmune liver disease, etc.) and neuropsychiatric symptoms from other causes; and (3) patients who were transferred or gave up treatment.

### Clinical and lab indexes

The indexes included age, gender, white blood cells (WBC), neutrophils (NEUT), red blood cells (RBC), platelets (PLT) and hemoglobin (HGB), alanine aminotransferase (ALT), aspartate aminotransferase (AST), alkaline phosphatase (AKP), albumin (ALB), uric acid (UA), serum calcium (Ca), serum phosphorus (P), and other related indicators.

### Scoring method

The scoring method was determined according to the improved Leipzig scoring criteria issued by the Indian National Association for Study of the Liver (INASL) (7), as shown in Table 1.

**Table 1:** Leipzig scoring system for diagnosis of Wilson's disease.

	0	1	2	3	4
KF rings	−		+		
Serum ceruloplasmin (mg/dl)	>20	11–20	6–11	0–5	
24 h urinary copper (mcg) (in the absence of acute hepatitis)	<40	40–100	>100		
Coombs-negative hemolytic anemia with liver disease	−	+			
Mutational analysis		1 Chromosome			2 Chromosomes
Liver biopsy for histology S/O WD (Orcein-or rhodamine positive granules)		+			
Neurobehavioral symptoms			+		
Typical features on MRI brain		+			
History of Wilson’s disease in a family member		+			

≥4 or more: diagnosis established; 3: diagnosis possible, more tests needed; ≤2: diagnosis very unlikely. WD, Wilson’s disease.

### Statistical analysis

The statistical analysis was performed using SPSS version 26.0 and R version 4.1.0 software. Variables with *p *< 0.1 in the univariate analysis were included in the multivariate analysis to determine the risk factors associated with WD. *p *< 0.05 was considered significant. SPSS software was used for the univariate analysis of categorical variables (such as gender) using the chi-square test, expressed as *n* (%). For the single factor analysis of continuous variables (such as age, AST, etc.), the Kolmogorov–Smirnov test was used to examine the normality, and a non-parametric test was used to analyze the non-compliance. If the indicators obey the normal distribution, then the use of independent sample T test analysis. The statistically significant indicators in the univariate analysis results were retained, and a multivariate binary logistic regression analysis was included to screen out the independent influencing factors of WD. R software was used to establish a nomogram prediction model for independent influencing factors. The nomogram prediction accuracy was evaluated and quantified by calculating the concordance index (C-index) and calibration curve. At the same time, the receiver operating characteristic (ROC) curve of the nomogram prediction model and the scoring system was drawn, and the area under the ROC curve (AUC) was calculated to compare the predictive ability of the nomogram model and the current Leipzig score for WD.

### Research methods

Patients with complete clinical data were included in the study. SPSS version 26.0 software was used for statistical processing to screen out independent predictors, establish a prediction model for the occurrence of WD, calculate the C-index, and draw a correction curve to evaluate and quantify the accuracy of the model. The ROC curve was used to compare the predictive ability of the prediction model and the related scoring system, and the results were analyzed and discussed.

## Results

### Univariate analysis of clinical data of Wilson's disease and non-Wilson's disease

A univariate analysis was performed based on the collected clinical data of patients, and independent influencing factors that may lead to WD in common indicators, such as blood routine, blood biochemistry, and related electrolytes, were included in this study. SPSS software was used for the univariate analysis of categorical variables (such as gender) using the chi-square test, expressed as *n* (%). The Pearson chi-square test and Fisher’s exact probability test were used for the comparison between the groups. For the single factor analysis of continuous variables (such as age, ALT, etc.), the Kolmogorov–Smirnov test was first used to examine its normality. Non-parametric test for analysis of disobedient. Normal distribution was examined using the independent sample *t*-test analysis, as shown in Table 2.

**Table 2:** Univariate analysis of clinical indexes in Wilson's disease.

Indexes	WD group (*n* = 127)	Non-WD group (*n* = 73)	*χ*^2^/*Z*	*P* value
Age (years)	23 (17–34)	27 (18–36)	−1.652	0.100
Gender [*n* (%)]				
Male	63 (49.60)	35 (47.95)	0.051	0.821
Female	64 (50.39)	38 (52.05)		
WBC (10^9^/L)	4.5 (3.26–6)	4.53 (3.3–6.4)	−0.435	0.663
NEUT (10^9^/L)	2.37 (1.78–3.51)	2.94 (1.95–3.73)	−1.494	0.135
RBC (10^12^/L)	4.25 (3.83–4.69)	4.21 (3.75–4.64)	−0.442	0.659
HGB (g/L)	124.24 ± 26.39	124.37 ± 17.76	0.039	0.007
PLT (10^9^/L)	108 (69–154)	110 (81–137)	−0.467	0.641
AST (U/L)	32 (19–79)	16 (12–28)	−6.335	<0.05
ALT (U/L)	23.0 (13.8–49.5)	18.0 (12.5–26.0)	−2.929	0.003
AKP (U/L)	130.5 (91.8–130.5)	82.0 (65.0–95.5)	−6.896	<0.05
ALB (g/L)	36.7 (30.4–40.1)	45.6 (44.3–47.5)	−10.60	<0.05
UA (μmol/l)	161.5 (123.8–228.3)	258.0 (234.0–321.0)	−7.48	<0.05
Ca (mmol/L)	2.14 (2.01–2.24)	2.26 (2.20–2.39)	−6.586	<0.05
P (mmol/L)	1.03 (1.03–1.03)	1.24 (1.22–1.32)	−11.07	<0.05

WD, Wilson’s disease; WBC, white blood cells; RBC, red blood cells; ALT, alanine aminotransferase; AST, aspartate aminotransferase; AKP, alkaline phosphatase; NEUT, neutrophils; PLT, platelets; ALB, albumin; UA, uric acid; Ca, serum calcium; P, serum phosphorus; HGB, hemoglobin.

In order not to omit meaningful variables, the inclusion criteria were relaxed to *p *< 0.1. The results showed that there was no significant difference in WBC, NEUT, RBC, and PLT between the two groups (*p *> 0.1). There were significant differences in AST, ALT, AKP, ALB, UA, Ca, P, and HGB between the two groups (*p *< 0.1). Based on the above single factor analysis results, AST, ALT, AKP, ALB, UA, Ca, P, HGB, and other indicators were selected as suspicious influencing factors of WD.

### Multivariate analysis of clinical data

Based on the above univariate analysis results, AST, ALT, AKP, ALB, UA, Ca, P, and HGB were included in the multivariate analysis. AST, ALT, AKP, ALB, UA, Ca, P, and HGB were used as independent variables, and WD was used as the dependent variable. A binary logistic multivariate regression analysis was used to screen independent predictors of WD. The results are shown in Table 3.

**Table 3:** Multivariate analysis of clinical indicators of Wilson's disease.

Indexes	*B*	SE	Wald	*P*	OR	95% CI of OR	
						Lower	Upper
AST	0.058	0.013	19.625	<0.01	1.059	1.033	1.087
ALT	0.036	0.011	10.539	0.001	1.036	1.014	1.059
AKP	0.028	0.007	17.764	<0.01	1.028	1.015	1.042
ALB	−0.594	0.090	43.530	<0.01	0.552	0.463	0.659
UA	−0.010	0.002	26.202	<0.01	0.990	0.987	0.994
Ca	−7.643	1.316	33.721	<0.01	0.001	0	0.006
P	−11.262	2.548	19.533	<0.01	0	0	0.002
HGB	0	0.006	0.001	0.969	1.00	0.988	1.012

ALT, alanine aminotransferase; AST, aspartate aminotransferase; AKP, alkaline phosphatase; ALB, albumin; UA, uric acid; Ca, serum calcium; P, serum phosphorus; HGB, hemoglobin.

In binary Logistic multivariate analysis, AST, ALT, ALP, ALB, UA, Ca, P, p-values were less than 0.05, the difference was statistically significant.

The odds ratio (OR) value of AST was 1.059, the influence coefficient B was 0.058, and the 95% confidence interval (CI) was 1.033–1.087, which indicated that patients with AST 1 unit higher were 1.059 times more likely to develop WD than those with AST 1 unit lower. The OR value of ALT was 1.036, the influence coefficient B was 0.036, and the 95% CI was 1.014–1.059, indicating that patients with ALT 1 unit higher were 1.036 times more likely to develop WD than those with ALT 1 unit lower. The OR value of AKP was 1.028, the influence coefficient B was 0.028, and the 95% CI was 1.015–1.042, indicating that the possibility of WD in patients with AKP 1 unit higher was 1.028 times that in patients with AKP 1 unit lower. The OR value of ALB was 0.552, the influence coefficient B was 0.09, and the 95% CI was 0.463–0.659, indicating that patients with ALB 1 unit lower were 0.552 times more likely to develop WD than patients with ALB 1 unit higher. The OR value of UA was 0.99, the influence coefficient B was −0.01, and the 95% CI was 0.987–0.994, indicating that the probability of developing WD in patients with UA 1 unit lower was 0.99 times that in patients with UA 1 unit higher. The OR value of Ca was 0.001, the influence coefficient B was −7.643, and the 95% CI was 0–0.006. The OR value of *P* was 0, the influence coefficient B was 11.262, and the 95% CI was 0–0.002, indicating that the higher the AST, ALT, and AKP, the higher the possibility of WD, and the lower the ALB, UA, Ca, and P, the higher the possibility of WD.

The results showed that AST, ALT, AKP, ALB, UA, Ca, and P could be used as independent risk factors to predict the occurrence of WD. Therefore, the above indicators are included in the construction of the prediction model as independent predictors.

### Construction of Wilson's disease nomogram prediction model

**Figure 1:**
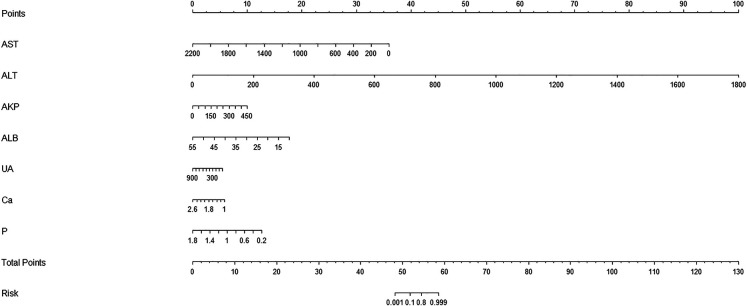
A nomogram prediction model of Wilson's disease.

Based on the rms package in R software, the independent predictors screened by the above multi-factor analysis were included to construct a nomogram prediction model for the occurrence of WD. As shown in Figure 1, the six predictors on the left side were AST, ALT, AKP, ALB, UA, Ca, and P, from top to bottom. According to the scores of different influencing factors corresponding to the actual clinical data of the patients, the scores of the six predictors were added to obtain the total score. The value obtained by making the total score vertically downward is the prediction probability of WD.

### Verification of Wilson's disease nomogram prediction model

**Figure 2:**
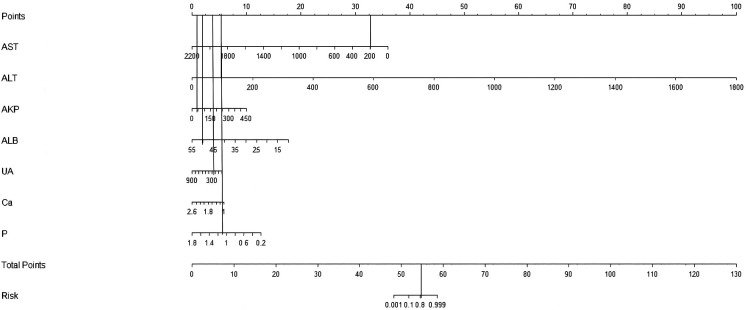
Application of nomogram prediction model for Wilson's disease.

For example, a patient underwent serum biochemical tests to obtain AST 200 U/L, ALT 100 U/L, AKP 50 U/L, ALB 50 g/L, UA 300 μmol/L, Ca 1.1 mmol/L, and P 1.0 mol/L. As shown in Figure 2, the patient had an AST score of 32, an ALT score of 5, an AKP score of 1.25, an ALB score of 2.5, a UA score of 3.75, a Ca score of 5, a P score of 5, and a total score of 54.5 based on the predictive model. When the total score was plotted vertically downward, the sum was about 80 %, indicating that the patient had a predicted probability of WD diagnosis of 80 %. This prediction model guides clinicians to make the next diagnosis and individualized treatment to reduce the probability of disease occurrence..

#### ROC curve of nomogram prediction model

**Figure 3:**
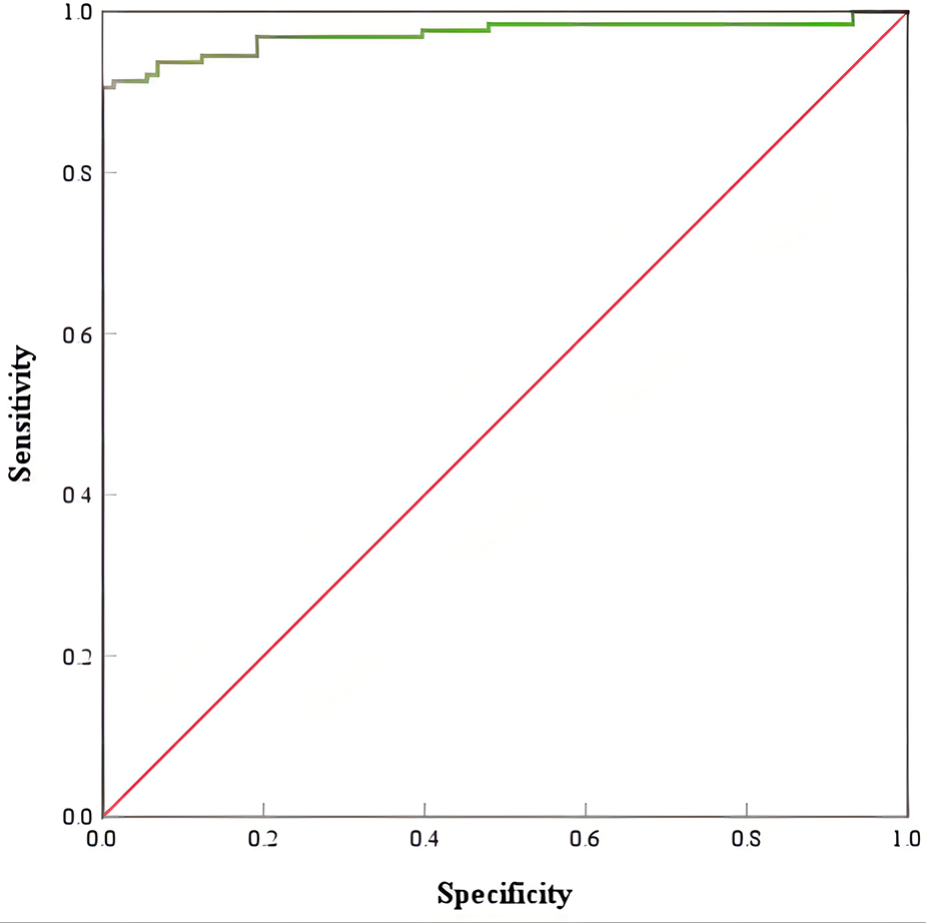
ROC curve of Wilson's disease prediction model. ROC, receiver operating characteristic.

The ROC curve of the nomogram prediction model is shown in Figure 3; the AUC was 0.971, the optimal threshold was 0.698, the sensitivity was 0.906, and the specificity was 1. The AUC has a predictive effect when the value is in the range of 0.5–1.0. The AUC value of the nomogram prediction model is 0.971 > 0.7, indicating that the prediction accuracy is good. The score above the optimal critical point of 0.698 can predict WD in high-risk groups, while a score lower than the optimal critical point of 0.698 can predict WD in low-risk groups. The corresponding sensitivity, specificity and 95% CI were 90.6%, 100%, and 0.948–0.995, respectively.

#### C-index and calibration curve

The Bootstrap self-sampling method was used to perform 1,000 random samplings to verify the prediction model. The accuracy of the WD prediction was evaluated by calculating the C-index and the calibration curve. The C-index can be used to evaluate the predictive ability of the model and predict the probability that the predicted results are consistent with the observed results. A C-index in the range of 0.5–1 indicates that the model has a predictive effect, while a C-index above 0.7 indicates that the ability to predict the model is more accurate. The C-index value of the constructed nomogram prediction model is 0.972, indicating that the prediction model has good accuracy.

**Figure 4:**
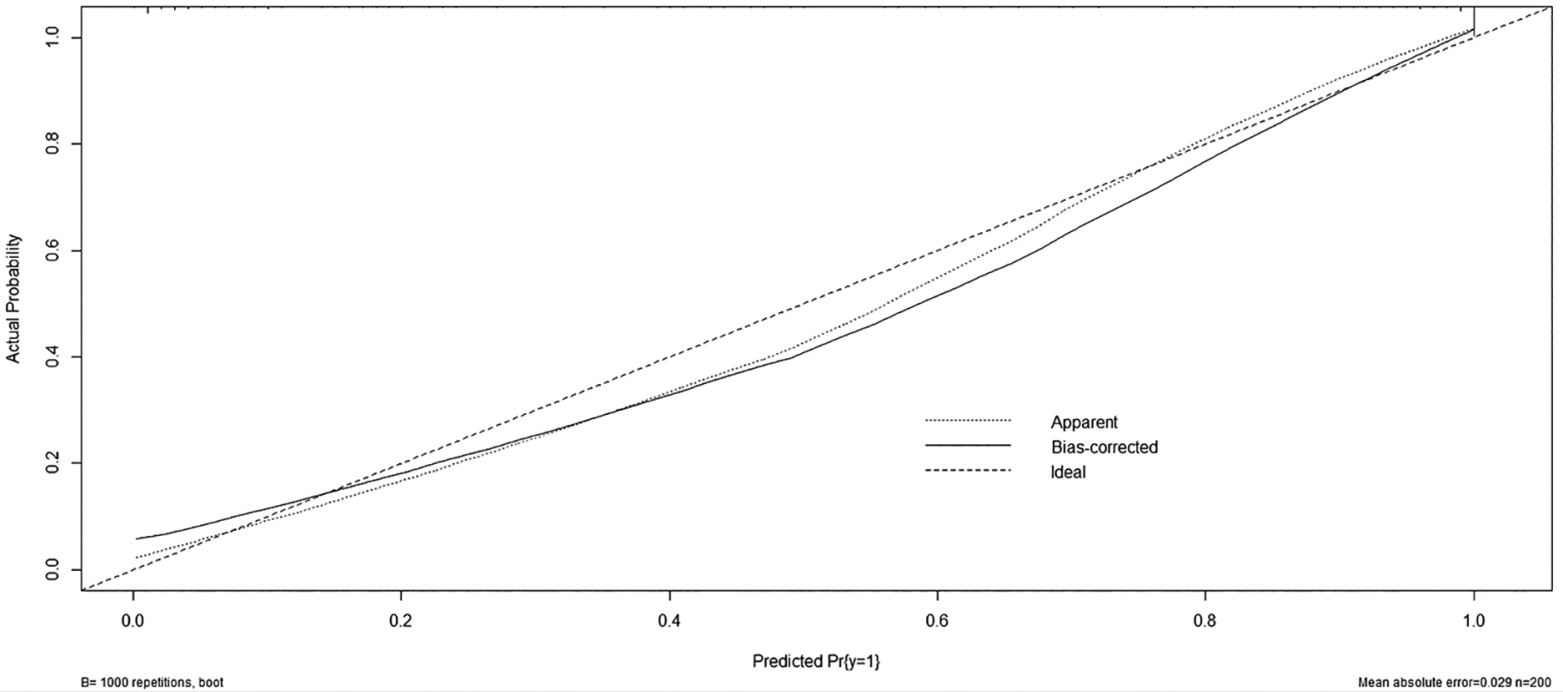
Wilson's disease prediction model calibration curve.

At the same time, the calibration curve is drawn, as shown in Figure 4; the dotted line represents the ideal curve corresponding to the predicted result and the actual result. If the predicted incidence of the calibration curve is closer to the dotted line, the prediction ability of the model is more accurate. The x-axis represents the predicted incidence of the nomogram, while the *y*-axis represents the actual incidence. The calibration curve is well fitted with the ideal curve, indicating that the nomogram prediction model has a good prediction ability on the occurrence of WD.

### Comparison of nomogram prediction model and related scoring prediction ability

The ROC curve of the WD nomogram prediction model and Leipzig score was drawn, and the AUC value was calculated, as in Table 4, to compare the predictive ability of the nomogram model and Leipzig score.

**Table 4:** AUC comparison of prediction models and Leipzig score for Wilson's disease

Variable	AUC	SE	*P*	95%CI	
				Lower	Upper
Prediction model	0.971	0.012	<0.01	0.948	0.995
Leipzig score	0.969	0.010	<0.01	0.949	0.988

AUC, area under the curve.

**Figure 5:**
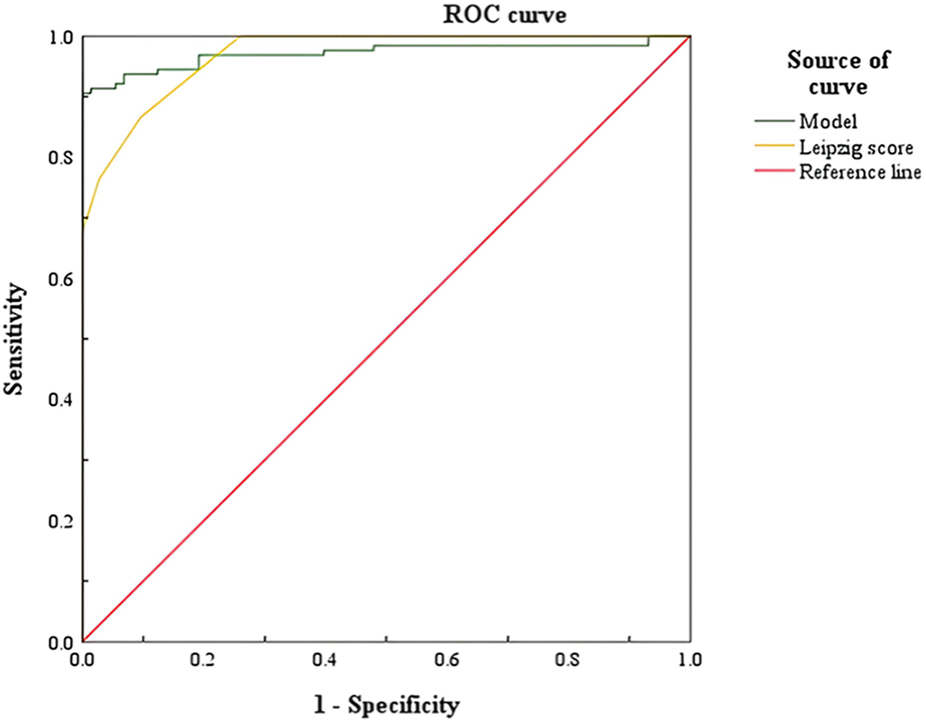
Comparison of ROC curves of Wilson's disease prediction model and Leipzig score. ROC, receiver operating characteristic.

The results show that the AUC of the prediction model is 0.971 and the AUC of the Leipzig score is 0.969. The nomogram prediction model and the scoring system have a predictive value, and the AUC value of the nomogram prediction model is greater than the AUC value of the Leipzig score, indicating that the nomogram prediction model has a better prediction ability than the Leipzig score, as shown in Figure 5.

As shown in Figure 5, the AUC of the prediction model and the Leipzig score is above the standard line, indicating that the above prediction model and scoring system have a predictive value, and the nomogram prediction model has a better predictive effect on WD.

## Discussion

The clinical symptoms of WD are not typical; in the early onset, a variety of symptoms appear, such as obvious damage to the liver and nervous system, kidneys, blood, and joint problems, and these can cause people's attention to the disease. However, in the early stage of the disease, especially in children, the clinical symptoms are not obvious, and some children with the disease have no symptoms in the liver and nervous system. The early diagnosis of the disease has a great impact on whether it can be well treated, and it is very important to reduce clinical misdiagnosis.

This study combined the inclusion and exclusion criteria to sort the clinical data of 200 patients in the First People's Hospital of Yunnan Province. Through a univariate analysis and multivariate binary logistic analysis, AST, ALT, AKP, ALB, UA, Ca, and P were selected as independent risk factors for WD. These seven independent risk factors were used to construct a nomogram prediction model. After verification, it was found that the nomogram prediction model had a better prediction effect, and its prediction ability was more accurate than the Leipzig score.

### Predictive value of each index for Wilson's disease

#### Predictive value of liver enzymes for Wilson's disease

Liver disease may be the first manifestation in 60% of patients with WD in all age groups. It may be completely asymptomatic, a coincidental finding, or signs and symptoms suggesting the presence of any form of liver disease. Progress may be slow or very rapid (8). The liver enzyme spectrum can be used as one of the serological indicators for the diagnosis of WD with liver injury, including AST and ALT, which can last for more than 6 months in asymptomatic patients (9). Clinically, it can be manifested as asymptomatic hepatomegaly, a continuous increase of liver function enzymes, acute or chronic hepatitis, compensatory or decompensated cirrhosis, and even fulminant liver failure. The disease usually progresses rapidly, and the possible forms of liver injury are as follows: (1) free copper ions in the blood directly damage the liver cell membrane, which may be related to the reduction of the intracellular glutathione level and glutathione reductase activity, aggravation of oxidative stress response, and activation of inflammatory response (10); (2) *in vitro* cell experiments showed that excessive copper can activate caspase-dependent and independent pathways to apoptosis, further aggravating the death of liver cells (11); (3) the gradual necrosis of liver cells to release a large number of copper ions, increasing the number of free copper ions in the blood, further aggravating liver injury; and (4) red blood cells in the blood are destroyed to produce a large number of metabolites, such as bilirubin, which can also aggravate the degree of liver damage.

In a cross-sectional study, 60 children aged 3–18 years with acute liver failure were divided into a WD acute liver failure (WALF) group and other causes of acute liver failure (non-WALF) group. The results showed that the mean HGB, mean ALT, mean AKP, and mean ceruloplasmin in WALF children were lower than those in the non-WALF group (12). No acute liver failure occurred in the patients included in this study, but the reduction of the above indicators can be used as a basis for further diagnosis of acute liver failure in WD. In this study, the AST level was 32 U/L in the WD group and 16 U/L in the non-WD group. The ALT level was 23 U/L in the WD group and 18 U/L in the non-WD group. There are significant differences in the liver enzymes between the two groups. A liver enzyme spectrum examination is feasible regardless of whether the patient has symptoms of liver injury, which is one of the predictors for the diagnosis of WD. The multivariate analysis of liver enzymes (AST, ALT) and the occurrence of WD were significantly correlated; *p *< 0.05, which is statistically significant, is an independent factor.

#### Predictive value of alkaline phosphatase for Wilson's disease

AKP is a part of the zinc metalloproteinase family, which catalyzes the hydrolysis of phosphate esters at an alkaline pH level. This enzyme exists on the tubular membrane of hepatocytes. Typically, AKP increases with bile duct obstruction, which is due to an increase in the synthesis of AKP in the tubules, which are then transferred to the blood sinus, also an indicator of liver injury (13). A study has shown that the ratio of AKP/TBIL < 4 and AST/ALT > 2.2 can be used as one of the bases for the disease (14). In this study, WD can cause damage to the hypothalamus–pituitary–peripheral target gland axis, a decreased parathyroid hormone, resulting in bone metabolism disorders, and thus elevated levels of AKP. The AKP level in the WD group was 130.5 U/L, and the AKP level in the non-WD group was 82 U/L. It can be seen that the two groups of AKP have significant differences. The results of the multivariate analysis confirmed that AKP was an independent predictor of WD (*p *< 0.05, with statistical significance).

#### Predictive value of albumin for Wilson's disease

ALB is one of the most important proteins in human body. ALB has many functions, including the maintenance of colloid osmotic pressure (COP) within the intravascular and extravascular body compartments and carrying endogenous hormones and ions, as well as exogenous drugs, scavenging oxygen-derived free radical species in sites of inflammation, participating in the acid–base balance within the body, contributing to waste product elimination, and mediating coagulation (15–17).

ALB is a liver synthetic protein, and its expression is related to the change of epithelial cell synthesis function in the liver. After a liver injury, the epithelial cells are partially apoptotic, which reduces the synthesis of ALB by epithelial cells, resulting in a decrease in its level. There are few reports on the changes of serum albumin index in patients with WD. In patients with WD, the liver is the first and most important accumulation organ of copper ions. Long-term accumulation can cause cirrhosis, liver synthesis and secretion function damage, protein synthesis, and secretion reduction. The kidney copper deposition can cause renal tubular damage, especially distal convoluted tubule damage caused by resorption disorders, such as excessive protein in urine, decreasing ALB. In this study, the ALB level in the WD group was 36.7 g/L and the ALB level in the non-WD group was 45.6 g/L. There was a significant difference between the two groups. The multivariate analysis confirmed that ALB was an independent predictor of WD (*p *< 0.05, with statistical significance).

#### Predictive value of uric acid for Wilson's disease

Renal function tests can determine whether there is kidney damage. Copper ions are filtered and reabsorbed when passing through the glomeruli and renal tubules. Excessive copper ions are deposited in the glomeruli and renal tubules, affecting renal function. The synthesis pathway of UA in vivo is mainly generated by a series of oxidation reactions of purine metabolism in the liver. The most important step is the three-step oxidation reaction of hypoxanthine nucleotide (IMP) in the liver to generate hypoxanthine, xanthine, and UA in turn (18). The metabolic pathway of UA in the human body is mainly excreted by kidney metabolism through urine, and some is excreted through feces and sweat (19).

The more severe the liver damage, the lower the enzyme activity. Impaired renal tubular reabsorption function, renal tubular epithelial cell damage, thickening of the basement membrane, disappearance of cell brush border, structural disorders affecting the urate transporter 1 (URAT1) transmembrane transport of serum UA, and increased mitochondrial density in the cytoplasm lead to decreased ATP synthesis by mitochondria, which in turn affects the active transport of UA (20). Therefore, the greater the degree of liver damage, the lower the enzyme activity will be, and the less UA will be produced. Therefore, the change of serum UA level can indirectly reflect the degree of liver function damage in patients with WD with liver injury. In this study, the UA level in the WD group was 161.5 μmol/L and the UA level in the non-WD group was 258 μmol/L. There was a significant difference between the two groups. The multivariate regression analysis showed that uric acid was *p *< 0.05, which was consistent with the conclusion of the literature and was significantly correlated with WD.

#### Predictive value of serum calcium and serum phosphorus for Wilson's disease

Serum calcium plays a central role in a wide range of basic functions. Its metabolism is regulated by three major transport systems: intestinal absorption; renal reabsorption; and bone turnover (21). Since copper can damage the proximal tubules of the kidney and lead to the loss of calcium, phosphate, amino acids, and proteins, it can cause the increase of calcium and phosphorus in urine, leading to a reduction in serum calcium and serum phosphorus.

At present, most of the studies predicting the occurrence of WD do not cover serum calcium and serum phosphorus indicators. In this study, indicators of serum calcium and serum phosphorus in most patients with WD are lower than the lower limit of normal values. Ca in the WD group was 2.14 mmol/L, ALB in the non-WD group was 2.26 mmol/L, P in the WD group was 1.03 mmol/L, and P in the non-WD group was 1.24 mmol/L. The two variables were significantly different between the two groups. The multivariate regression analysis showed that the *p*-values of serum calcium and serum phosphorus were <0.05, which was significantly correlated with WD.

### Predictive value of nomogram prediction model for Wilson's disease

In this study, AST, ALT, AKP, ALB, UA, Ca, and P were selected as independent risk factors for WD by univariate analysis and multivariate binary logistic analysis. The above indicators were used to construct a nomogram prediction model to help determine a diagnosis of WD, aiming to provide clinical guidance for clinicians and promote the process of precision medicine.

The nomogram prediction model is a visual and intuitive statistical model that can be used as a clinical tool to provide predictive information for clinicians. In this study, the above indicators can be obtained by a routine serum biochemical examination, which has the advantages of good objectivity, quantifiable and long-term tracking, and provides a theoretical basis for clinicians to diagnose WD. By drawing the ROC curve of the nomogram, the AUC was 0.971. The best critical point is 0.698; anything greater than the best critical point of 0.698 can predict WD, that is, the high-risk population, while anything lower than the best critical point of 0.698 can predict non-WD, that is, the low-risk population. The corresponding sensitivity, specificity, and 95% CI were 90.6%, 100%, and 0.948–0.995, respectively. The nomogram prediction model and the ROC curve of the Leipzig score were drawn, and the AUC was calculated. The AUC value compares the predictive ability of the nomogram prediction model and the existing Leipzig score for WD. The AUC of the score is 0.969, indicating that the above prediction model and scoring system have a predictive value, and the AUC value of the prediction model is the highest, confirming that the nomogram prediction model has a better prediction ability.

Chen et al. used EmpowerStats software to predict patients with WD cirrhosis, including blood routine, 24-h urine copper, and serum ceruloplasmin in 346 patients with WD. It was discovered that the top five important predictors were platelet large cell count (P-LCC), red cell distribution width CV (RDW-CV, CV means corpuscular volume), serum ceruloplasmin, age at diagnosis, and mean corpuscular volume (MCV) (22).

In this study, a prediction model of WD was established, and seven indicators that are easily available in clinical practice were used as predictors. In terms of verification, the consistency index calculated by the Bootstrap self-extraction method was 0.972, indicating that the prediction model has good accuracy. The calibration curve was drawn, and the calibration curve was well fitted with the ideal curve, indicating that the nomogram prediction model had a good prediction effect on the occurrence of WD, and the sensitivity and specificity of the nomogram prediction model were high, which confirmed that the nomogram prediction model had a high clinical application value.

During a clinical diagnosis, the prediction of WD should be based on specific clinical data and actual conditions, and various scoring criteria and nomogram prediction models should be integrated.

## Limitations and deficiencies

Although the WD nomogram prediction model constructed in this study has been verified to have a good prediction effect, it still has its shortcomings and limitations. First, the study was a retrospective study, which meant selection bias and recall bias exist. Second, this study is verified by the Bootstrap self-extraction method, so the nomogram prediction model constructed in this study still needs further external verification and analysis to improve its accuracy. Third, the prediction model constructed by machine learning is a black box, which could not determine the weight of each variable. Finally, the WD group and non-WD normal control group in the study had certain limitations; the significant difference between the two groups of enzyme elevation is not surprising. If conditions permit, the establishment of a nomogram prediction model for WD and non-WD, but combined with other liver diseases such as hepatitis and cholestasis, will be carried out in the future, which can make our conclusion more accurate and is a good and reliable early diagnosis model for WD.

With the development of individualized treatment, the diagnosis and treatment models for WD may be different, so the prediction model may be biased. With the inclusion of large sample data, more accurate clinical prediction models for WD may continue to emerge.

## Conclusion

This study confirmed that—for the predictive diagnosis of WD—AST, ALT, AKP, ALB, UA, Ca, and P can effectively predict the occurrence of the disease. The model has certain clinical application value, and its specific value determination and effectiveness still need further prospective and large sample studies.

## Data Availability

The original contributions presented in the study are included in the article/Supplementary Material, further inquiries can be directed to the corresponding author.
